# Augmentation Rhinoplasty Using Iliac Crest Graft in Saddle Nose Deformity

**DOI:** 10.7759/cureus.9705

**Published:** 2020-08-12

**Authors:** Waleed M Alshehri, Abdullah Aldosari, Ahmed H Alherz, Omar A Alrashood, Bandar Al Qahtani

**Affiliations:** 1 Otolaryngology, King Saud Medical City, Riyadh, SAU; 2 Otolaryngology - Head and Neck Surgery, King Saud Medical City, Riyadh, SAU

**Keywords:** reconstruction, iliac crest, autograft, saddle nose

## Abstract

Autologous bone graft is one of the management solutions for saddle nose deformity. This helps from both functional and esthetic perspective. Several features of autologous graft specify the best material used to repair bony and cartilaginous nasal defects. This article describes a case of a female who presented with saddle nose deformity after incidental insertion of a button battery in her nostril during childhood which was followed by depressed nasal dorsum. It was managed with good outcomes by augmentation rhinoplasty using an iliac crest bone graft.

## Introduction

Rhinoplasty is a procedure for the reconstruction of the anatomical features of the nose into a more satisfying shape which depends directly on the surgeon's ability to achieve a high-quality modification [1]. Nasal foreign bodies frequently present during the first decade when the child seeks to discover the world by the nose [2]. Accidental intranasal button batteries insertion can lead to severe nasal deformity among children [3]. Structural restoration is taken into consideration when the nasal deformity includes both cartilaginous and bony structures [4]. Several features of autologous graft specify the best material used to repair bony and cartilaginous nasal defects, for instance, biocompatibility, low infection susceptibility, and cost-effectiveness [4]. Various procedures for corrective nose deformity have been described in the literature [5]. Autogenous bone is a possible source of hard structural grafts. Iliac crest bone graft provides a very effective benefit in case of serious saddle nose deformity [6].

## Case presentation

**Figure 1:**
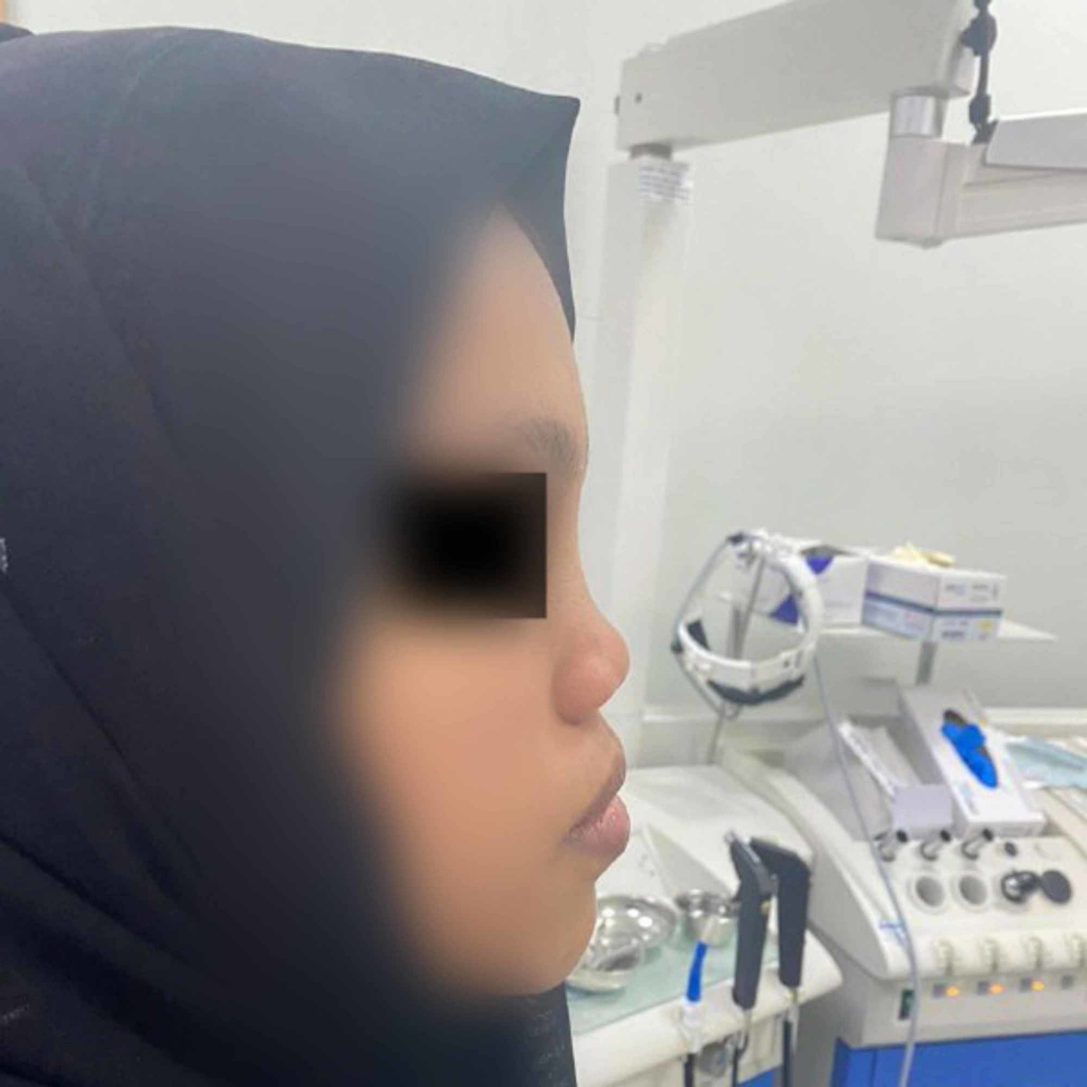
Preoperative lateral view of the patient.

**Figure 2:**
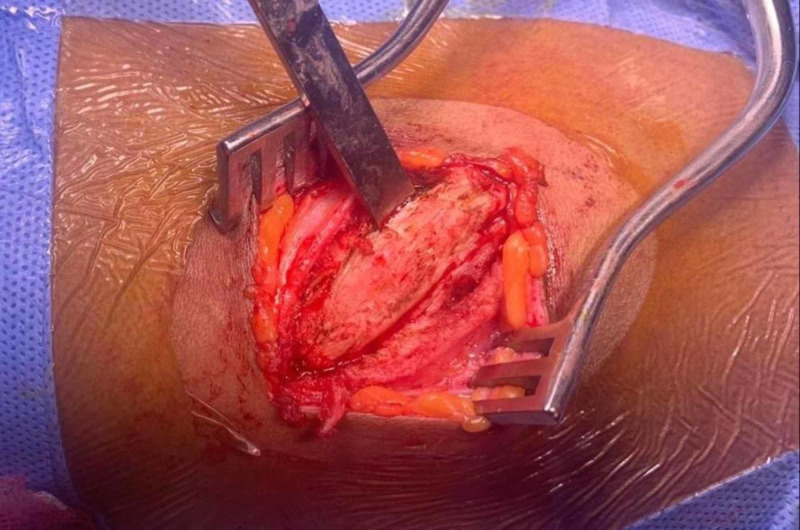
Iliac crest exposed and ready to harvest.

**Figure 3:**
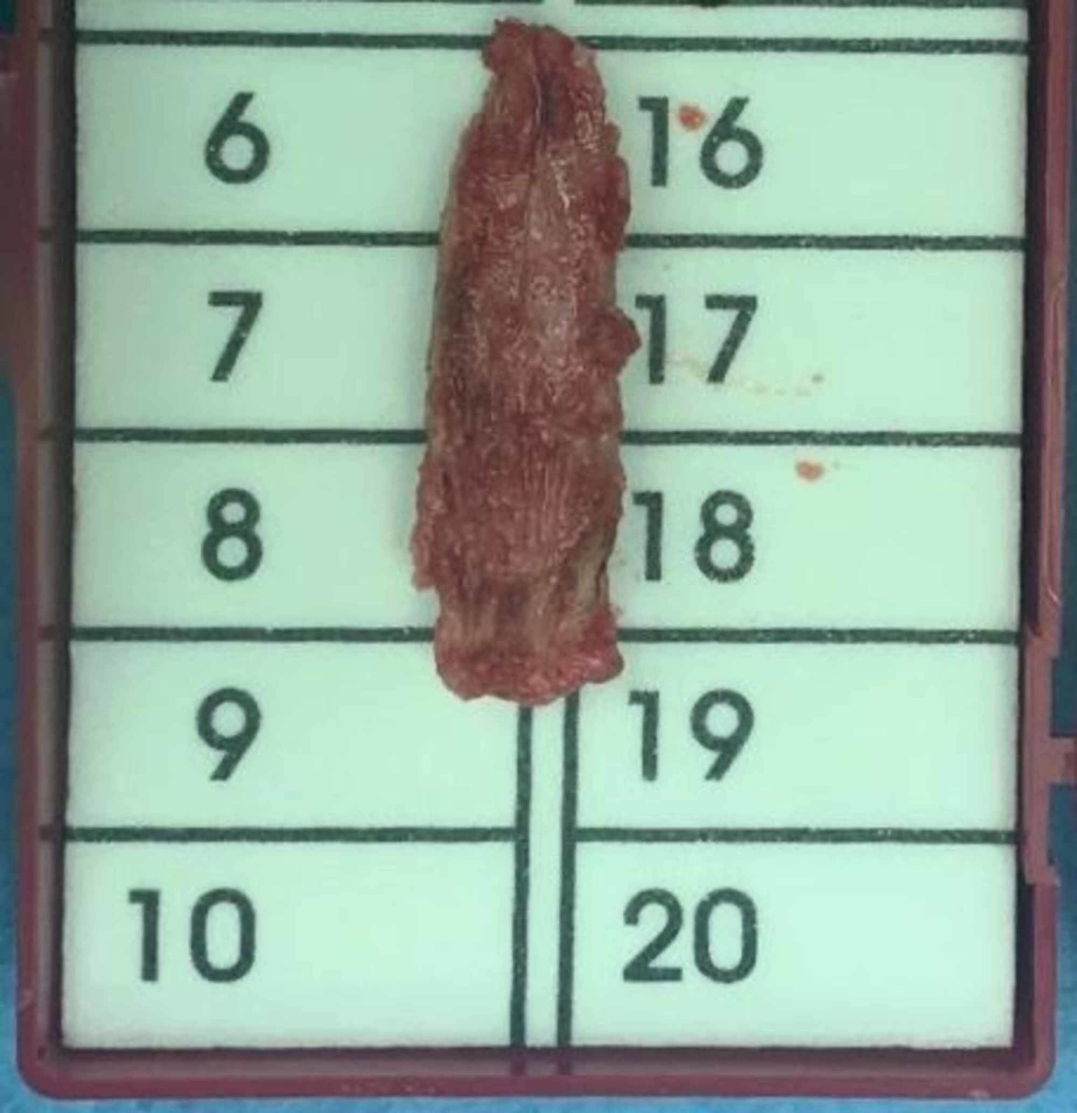
Iliac crest graft harvested.

**Figure 4:**
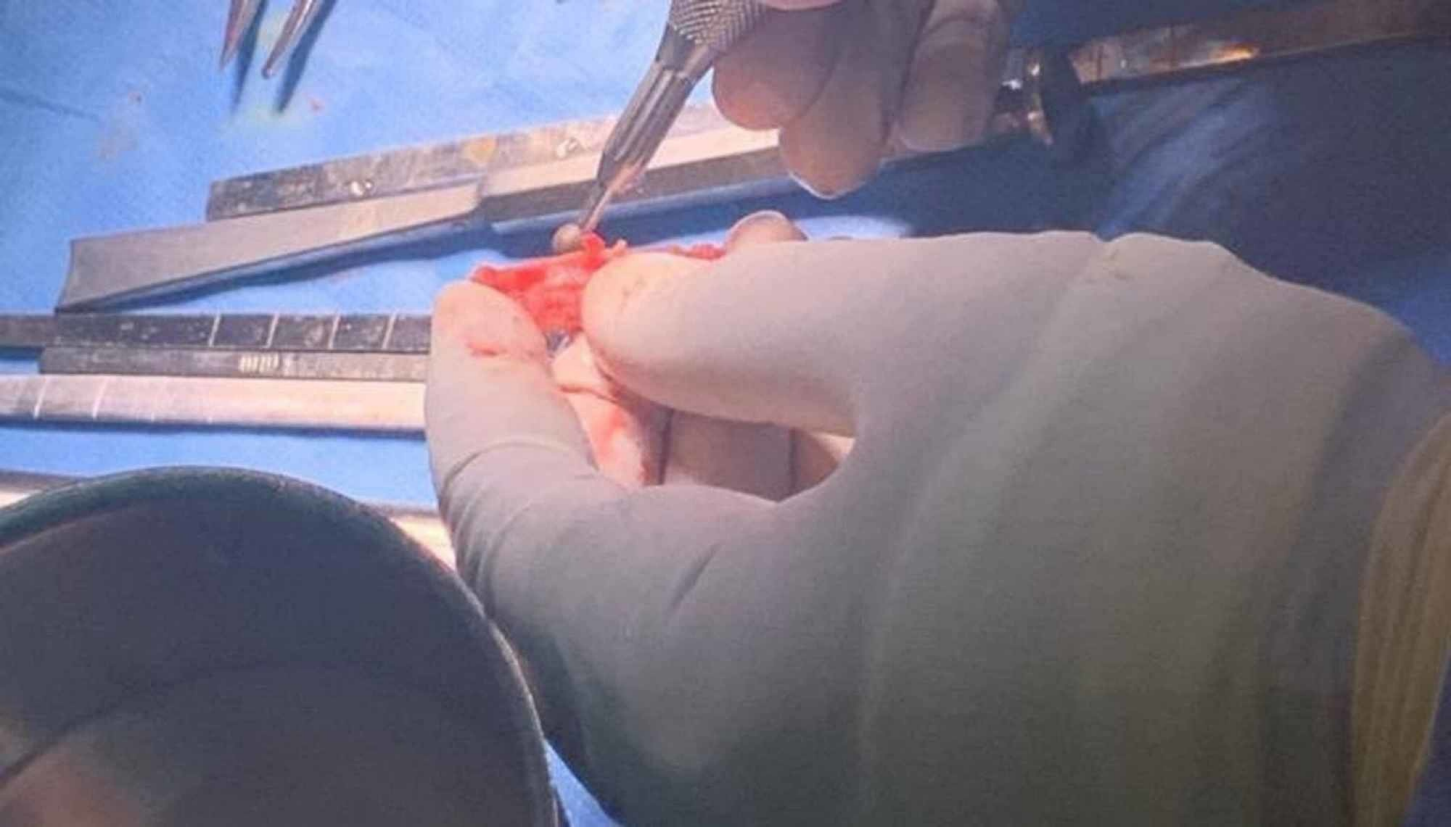
Reshaping and thinning the graft.

**Figure 5:**
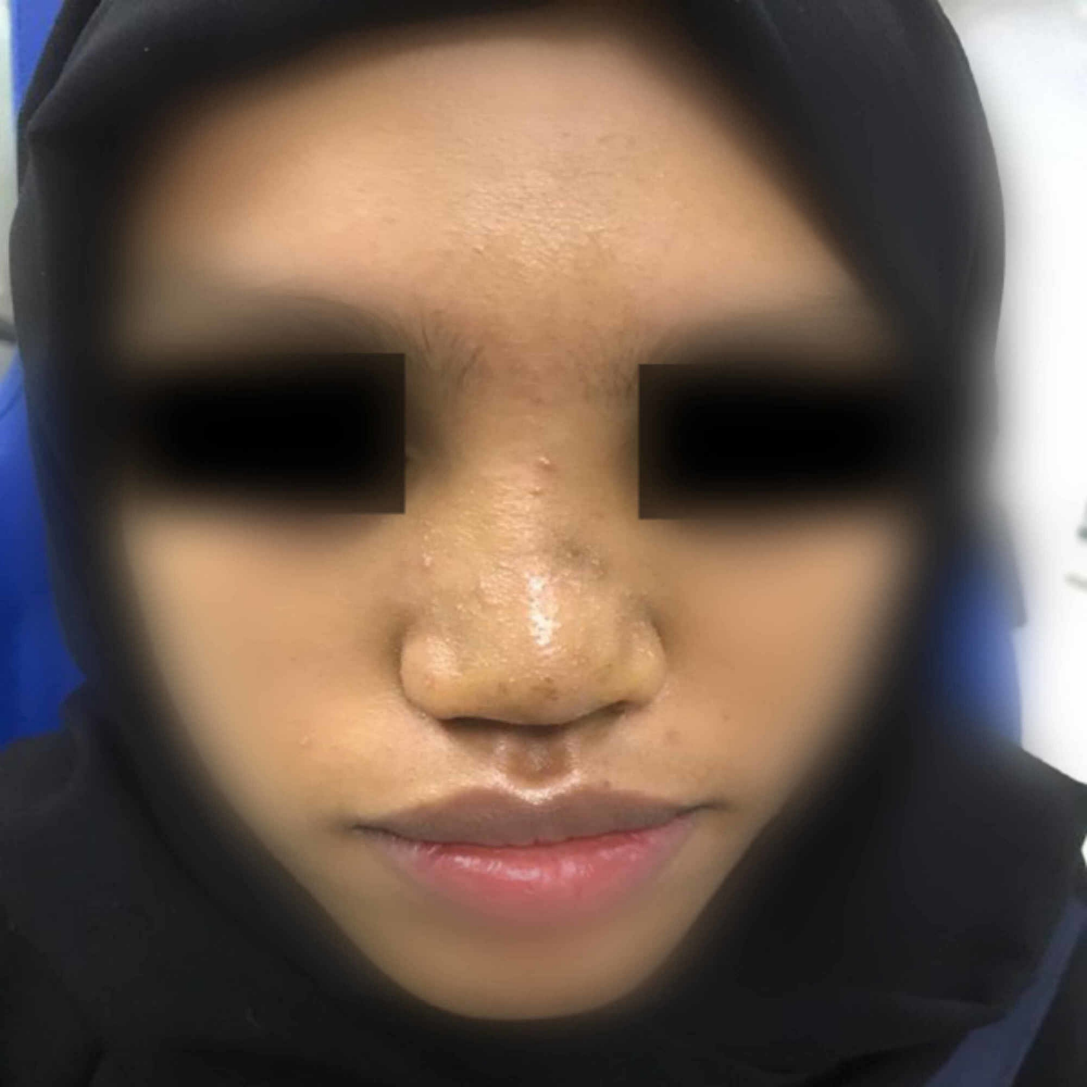
Postoperative anterior view of the patient.

**Figure 6:**
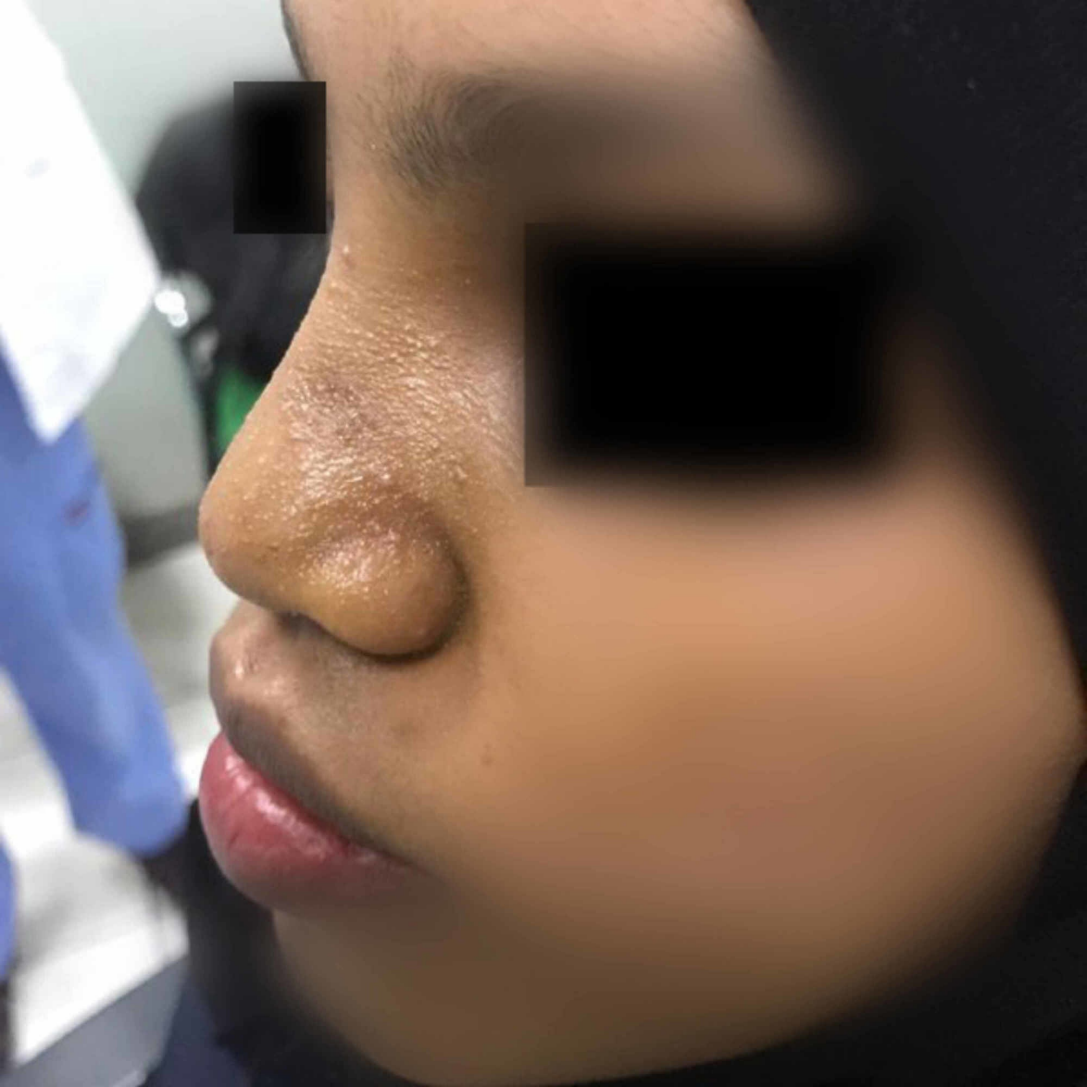
Postoperative lateral view of the patient.

We report a case of a 20-year-old female medically free who presented with the complaints of depression in her nose since childhood (Figure 1). She gave a history of incidental insertion of a button battery in her nostril at age five which was followed by depressed nasal dorsum. The battery was removed by her parents at that time. On general examination the patient was vitally stable, afebrile, and breathing in comfort. External nasal examination showed severe saddling and internal examination showed multiple additional tissue and both bony and cartilaginous septum were eroded. The patient consented for autograft from the iliac crest bone along with a proper explanation of the advantages and disadvantages of the procedure. Preoperative CT was done to evaluate her condition followed by taking her to the theater. Open rhinoplasty approach was carried out and in that same time the iliac crest graft was harvested by the assistant; then it was fashioned using a drill to smooth and even the edges (Figures 2-4). Graft was inserted and fixed followed by closing the skin of both the nose and the donor site. After five days from the procedure, the sutures were removed, and after six weeks the patient was satisfied with the surgical outcome (Figures 5-6).

## Discussion

Management of saddle nose deformity must be based on recognition of the pathological factors and classification of each case into curable pathology treated with septum straightening, absent nasal septum which required reconstruction, or as a result of the low dorsum with intact septum that treated with dorsal grafts [7]. A variety of grafting materials have been used for reconstruction -- autogenous bone graft (e.g., calvarium, rib, iliac crest) and cartilage graft (e.g., auricular conchal, costal) and easily manipulated alloplastic materials such as nonporous (e.g., silicone) and porous (e.g., porous polyethylene) [4]. Cartilaginous grafts are usually used in small deformities, however, large deformities are corrected through a bone graft [4]. Reconstruction of complicated nasal defect requires a more rigid structure for septum support and long-lasting outcomes [8]. Bone autograft in saddle nose places the septum to a midline position that helps from both functional and esthetic perspective [9]. Iliac crest bone graft is accessible in large amounts and it is very helpful for extreme cartilaginous and bony deformity, but difficult to reshape [1]. Rhinoplasty with iliac crest bone graft achieves excellent results with limited complications and serves particularly for significant saddling [6]. Successful reconstruction aimed to establish a stable, functional, and anatomically sound framework. Understanding the rigid and semi-rigid structures plays an important role in the perfect reconstruction of the nose [4]. Harvesting the anterior iliac crest bone graft is recommended as it is associated with a remarkable low risk of complications postoperatively [1]. However, it is a favored donor site associated with a 90% success rate and a 10% failure rate [1]. The iliac crest that is used in our case is one of the ideal bone grafts used to restore the nasal defect and enhance topographical irregularities [4].

## Conclusions

Augmentation rhinoplasty with iliac crest bone autograft is a valid option. It can achieve very acceptable results. Iliac crest autograft shows considerable patient satisfaction from an esthetic point of view after severe nasal saddling. It is one of the recommended bone grafts for the reconstruction of the complicated nasal defect that provides a rigid structure for the nasal septum support and long-lasting outcomes. Iliac crest bone graft is associated with a low risk of postoperative complications.
